# Quality and reliability of short videos about keloid on TikTok: A cross-sectional study

**DOI:** 10.1097/MD.0000000000049361

**Published:** 2026-06-19

**Authors:** Honggang Li, Qinyuan Wang, Xuanfen Zhang

**Affiliations:** a Department of Medical Aesthetic Surgery, The First Affiliated Hospital of Northwest University, Xi’an, Shaanxi, China; b Department of Plastic Surgery, Jinan Maternity and Child Care Hospital Affiliated to Shandong First Medical University, Jinan, Shandong, China; c Department of Plastic Surgery, The Second Hospital and Clinical Medical School, Lanzhou University, Lanzhou, Gansu, China.

**Keywords:** health information quality, keloid, short video, social media, TikTok

## Abstract

TikTok and similar short-form video services are now widely adopted as prominent platforms for circulating health knowledge. However, the quality of content on keloid remains unclear. Keloid, a pathological scar with significant physical and psychological impact, necessitates accurate public education. This cross-sectional study analyzed 122 keloid-related TikTok videos on February 9, 2026, using an unlogged search to minimize bias. Video quality was assessed with 3 scoring systems: the global quality score, the modified DISCERN, and the benchmark criteria of the Journal of the American Medical Association, and uploader categories, content themes, and engagement metrics were analyzed. Videos featured a median length of 65.5 seconds and strong user engagement, but holistic quality was modest (median global quality score = 3.0, modified DISCERN = 2.0, Journal of the American Medical Association = 3.0). Content predominantly covered treatment (86.1%) and clinical manifestations (52.5%), whereas etiology, diagnosis, and recurrence were underrepresented. Videos from plastic surgeons and healthcare professionals had significantly higher quality scores than those from individual users (*P* < .05). There was no relationship found between engagement metrics and quality. In conclusion, keloid-related TikTok videos achieve wide reach but have limited informational quality, emphasizing the need for enhanced professional involvement and more comprehensive content to improve educational value.

## 1. Introduction

Keloid formation represents a pathologic fibroproliferative disorder in which dense collagen accumulation extends well past the borders of the original cutaneous injury. Such lesions often detract from cosmetic appearance and elicit discomfort, pruritus, and dyschromia, contributing to a heavy physical and emotional burden. Managing this condition continues to pose clinical difficulty because of its erratic behavior and frequent relapse, even following surgical excision or adjuvant therapy. Epidemiological data consistently show pronounced interethnic disparities in keloid prevalence, pointing to a robust genetic predisposition underlying susceptibility.^[1,2]^ Keloid pathogenesis is driven by an intricate network of genetic factors, immune dysregulation, and defective wound repair, culminating in excessive collagen deposition and scar overgrowth.^[3–5]^ Although a substantial body of research has been devoted to this condition, the core molecular pathways driving keloid formation remain largely unresolved. Consequently, delivering dependable health content to the public now forms a fundamental component of strategies aimed at preventing and treating keloid. The information patients encounter during evaluation, diagnosis, and treatment – including educational content – plays a pivotal role in guiding their pursuit of medical care and the selection of optimal treatment options. The professionalism and accuracy of such information directly influence patients’ treatment decisions.

Recently, social media platforms – particularly short-form video services like TikTok – have gained prominence as powerful vehicles for health promotion, leveraging massive interactive user bases to disseminate reliable content across broad populations.^[6,7]^ These platforms’ algorithm-driven, high-velocity content delivery enables swift retrieval of health information, thereby consolidating TikTok’s role as an essential medium for public health communication.^[8,9]^ Concerns persist, however, regarding the reliability and quality of health-related content disseminated via such platforms. TikTok, referred to as Douyin in China, boasts a massive user base, and numerous content creators share keloid-related videos, which are subsequently recommended to users based on search behaviors and platform algorithms.^[10]^ However, due to the limited expertise of some creators, the reliability of these videos in guiding nonprofessionals regarding keloid diagnosis and treatment has not been rigorously evaluated. The quality of such content remains unverified, hindering public health education efforts.^[11,12]^

With the escalating incidence of keloid among Chinese individuals and the growing dependence on digital platforms for medical guidance, examining the current state of keloid-focused short videos on Douyin has become imperative.^[13]^ This investigation examined the distribution, quality, and credibility of keloid-focused videos on Douyin, compared content across uploader categories, and assessed how engagement metrics relate to informational value, aiming to advance the platform’s constructive role in health education and equip the public with reliable knowledge about keloid.

## 2. Methods

### 2.1. Ethics approval

This study did not enroll human participants, utilize clinical records, involve laboratory animals, or conduct histological analyses. All data used in this study were sourced from publicly available TikTok videos, and data collection activities fully complied with TikTok’s service agreements. No personal or identifying information was gathered or analyzed, and no communication with video creators took place. As such, institutional ethics committee approval was not required for this work.

### 2.2. Search strategy and data extraction

**Figure 1.**
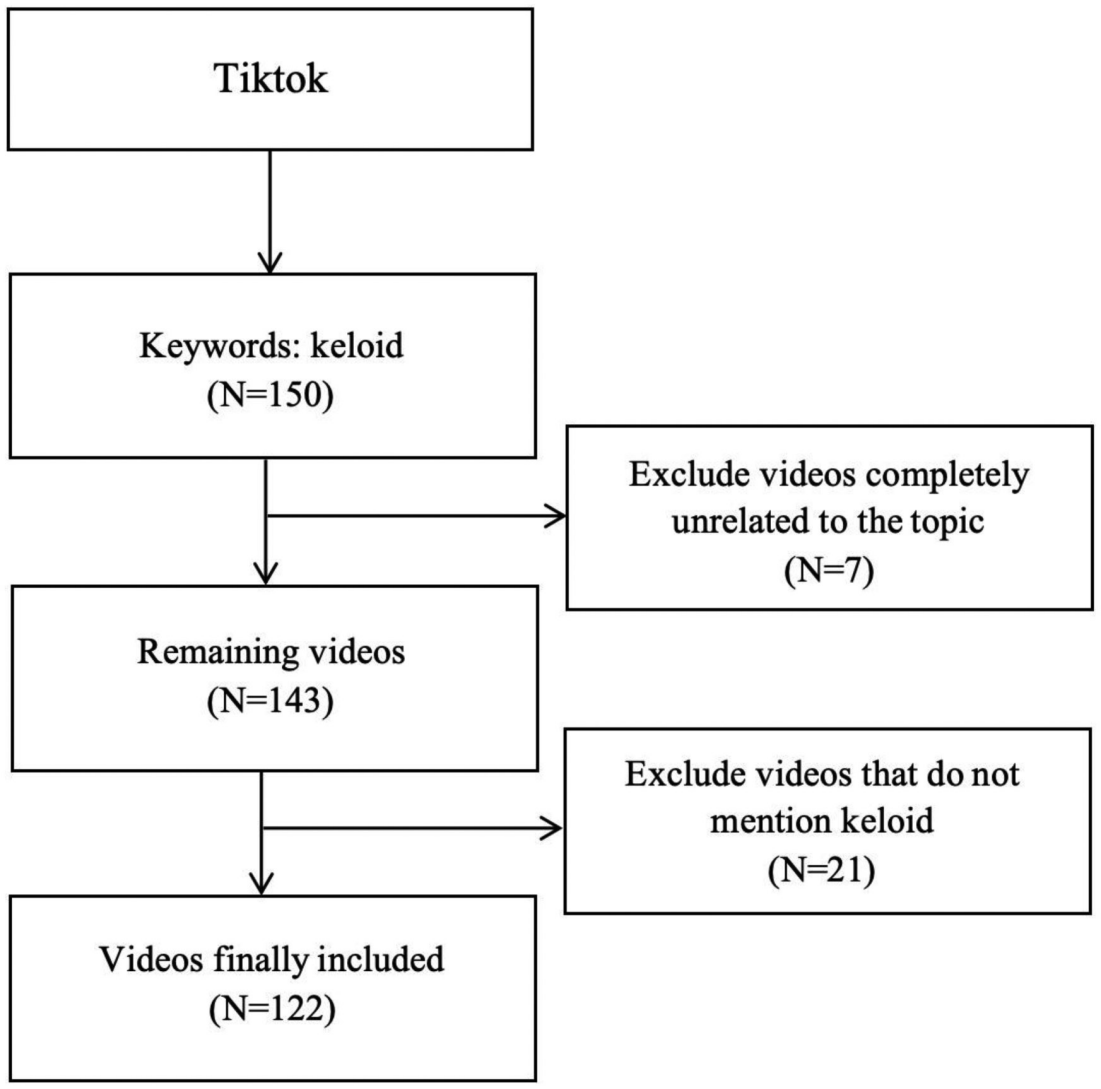
Flow chart of video selection.

To reduce analytical bias when assessing newly posted clips, we searched TikTok for the term “keloid” on February 9, 2026. We also performed searches without logging into any account, so as to rule out impacts from prior queries and algorithm-driven personalized suggestions. After the keyword search, we applied the platform’s native sorting rule to retrieve the first 150 entries. This study only enrolled videos concerning keloid. Any materials inconsistent with the subsequent standards were eliminated: unrelated to the topic or not mentioning keloid; consisting only of images or screenshots without explanatory content; repetitive content; and advertisements. Screening rules for enrollment and exclusion were set before content retrieval and data gathering. Afterwards, a total of 122 eligible clips underwent thorough assessment (Fig. 1). To facilitate further research, detailed information of all analyzed videos was collected, such as titles, duration, view count, likes, shares, comments, and video sources. Based on the uploader’s background, videos were categorized as being from plastic surgeons, dermatologists, other healthcare professionals (OHPs), or individual users.

### 2.3. Quality and reliability assessment

Throughout the study, a clinician took charge of collecting clips and documenting related information. Two medical staff members performed quality scoring and reconciled results through communication. Discrepancies between the 2 main raters were resolved by a senior medical expert with professional seniority.

We adopted 3 assessment tools to grade content quality: the global quality score (GQS), the modified DISCERN (mDISCERN) scale, and the Journal of the American Medical Association (JAMA) benchmark criteria.^[14–17]^ This 5-level rating framework of GQS runs from 1 to 5 points, corresponding to gradual improvements in content standard. The mDISCERN scale evaluates 5 distinct aspects, namely, clarity, relevance, traceability, robustness, and impartiality, capped at 5 points. Four major indicators are checked under JAMA standards: authorship, source attribution, content timeliness, and conflict disclosure, with overall scores spanning 0 to 4. Specific assessment guidelines can be found in Tables 1 to 3.

**Table 1:** The global quality score quality criteria.

Item features	Points
Poor quality; poor flow of the videos; most information missing; not at all useful for patients	1
Generally poor quality; some information listed, but many important topics missing; of very limited use to patients	2
Moderate quality; suboptimal flow; some important adequately discussed, but other information poorly discussed; somewhat useful for patients	3
Good quality and generally good flow; most of the relevant information listed, but some topics not covered; useful for patients	4
Excellent quality and flow; very useful for patients	5

**Table 2:** The modified DISCERN quality criteria.

Reliability score
1. Is the video clear, concise, and understandable?
2. Are valid sources cited?
3. Is the content presented balanced and unbiased?
4. Are additional sources of content listed for patient reference?
5. Are areas of uncertainty mentioned?

**Table 3:** The Journal of the American Medical Association benchmark criteria.

Score	Score component	
1 score	Authorship	Author and contributor credentials and their affiliations should be provided.
1 score	Attribution	Clearly lists all copyright information and states references and sources for content.
1 score	Currency	Initial date of posted content and subsequent updates to content should be provided.
1 score	Disclosure	Conflicts of interest, funding, sponsorship, advertising, support, and video ownership should be fully disclosed.

### 2.4. Statistical analysis

We adopted descriptive statistics to organize continuous data. Non-normally distributed data are reported as median and interquartile range (IQR), while categorical data are shown via counts and percentages. For between-group comparisons, the independent *t* test was applied to normally distributed data, and the Mann-Whitney U test for data deviating from normality. The Kruskal-Wallis *H* test was chosen to compare 3 or more subgroups. Post hoc pairwise contrasts were carried out with the Dunn test once overall significance was detected. Spearman correlation analysis was used to explore associations between quality ratings (GQS, mDISCERN, JAMA) and interaction indicators, including likes, comments, shares, and favorites. All statistical tests adopted a two-sided threshold, with *P* < .05 defined as statistically significant. All computations were completed in R (version 4.5.2; R Foundation for Statistical Computing).

## 3. Results

### 3.1. Baseline data of keloid-related videos

The included videos were all uploaded after 2025. The median video duration was 65.50 seconds (IQR: 44.25–105.50). The highest number of likes for an included video was 16,104, the lowest was 17, and the median was 208.00 (IQR: 87.50–545.50). Audience interaction was limited: median values reached 24 comments (IQR: 8.00–100.25), 64 saves (IQR: 26.50–213.50), and 75 shares (IQR: 24.00–242.25). Video quality remained suboptimal, with median GQS, mDISCERN, and JAMA scores of 3.00 (IQR: 2.00–3.00), 2.00 (IQR: 2.00–2.00), and 3.00 (IQR: 2.00–3.00), respectively. Table 4 provides complete details.

**Table 4:** General characteristics, quality, and reliability scores of keloid-related videos on TikTok.

Variables	TikTok (n = 122)
General information	
Video length(s), M (Q1, Q3)	65.50 (44.25, 105.50)
Likes, M (Q1, Q3)	208.00 (87.50, 545.50)
Collections, M (Q1, Q3)	64.00 (26.50, 213.50)
Comments, M (Q1, Q3)	24.00 (8.00, 100.25)
Shares, M (Q1, Q3)	75.00 (24.00, 242.25)
Video content	
Etiology	43 (35.25%)
Clinical manifestation	64 (52.46%)
Diagnosis	36 (29.51%)
Treatment	105 (86.07%)
Prognosis	46 (37.70%)
Recurrence	31 (25.41%)
Video quality	
GQS score, M (Q1, Q3)	3.00 (2.00, 3.00)
mDISCERN score, M (Q1, Q3)	2.00 (2.00, 2.00)
JAMA score, M (Q1, Q3)	3.00 (2.00, 3.00)

GQS = global quality score, JAMA = Journal of the American Medical Association, mDISCERN = modified DISCERN.

### 3.2. Uploader information for keloid-related videos

**Figure 2.**
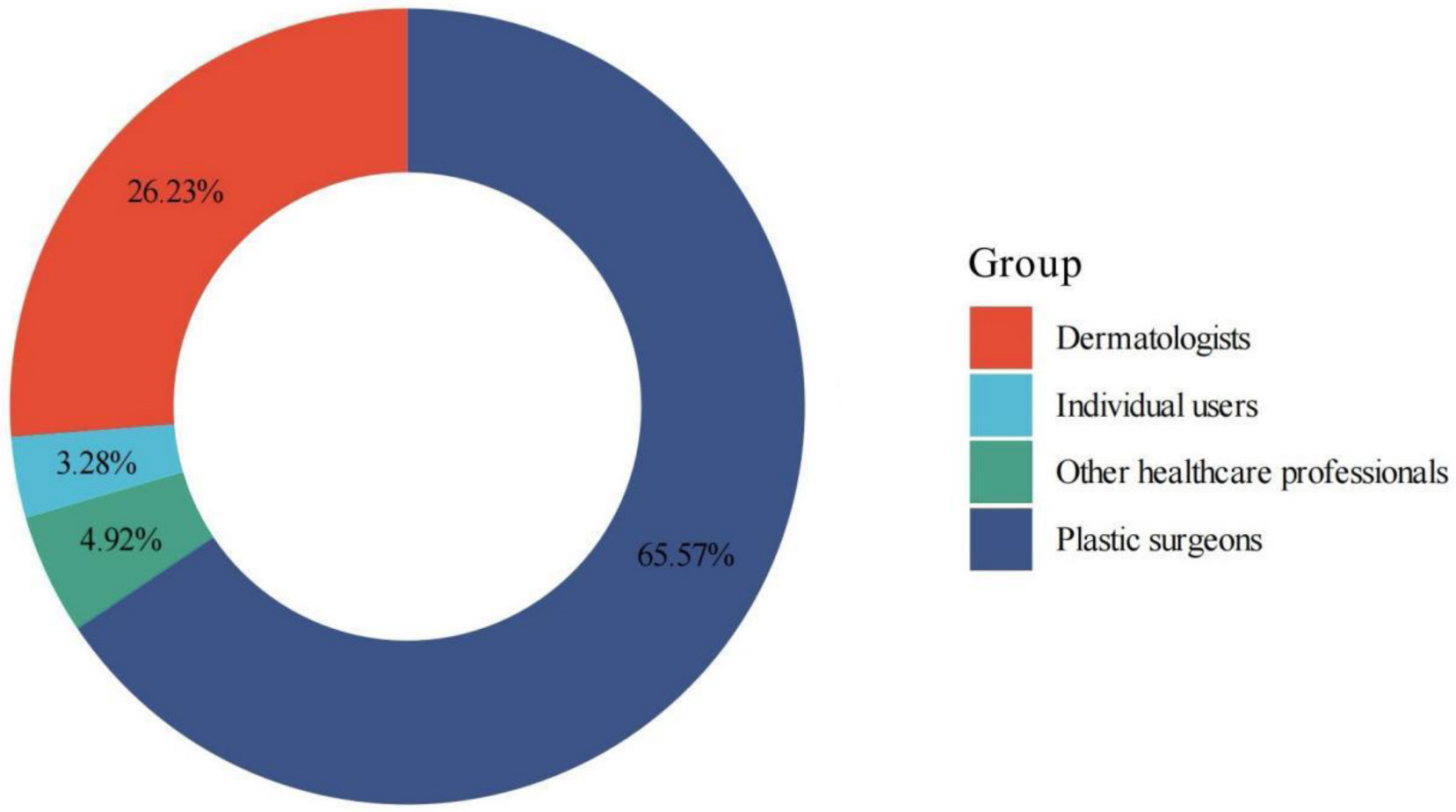
Breakdown of video contributors.

Plastic surgeons and dermatologists constituted the 2 most common uploader categories for keloid-related content (Fig. 2). Table 5 presents detailed information on the video sources.

**Table 5:** Video uploader categories.

Uploader	Number (%)
Plastic surgeon	80 (65.57%)
Dermatologist	32 (26.23%)
Hospital or department	3 (0.25%)
Nuclear medicine physician	2 (0.16%)
Burn surgeon	1 (0.08%)

### 3.3. Content of videos related to keloid

**Figure 3.**
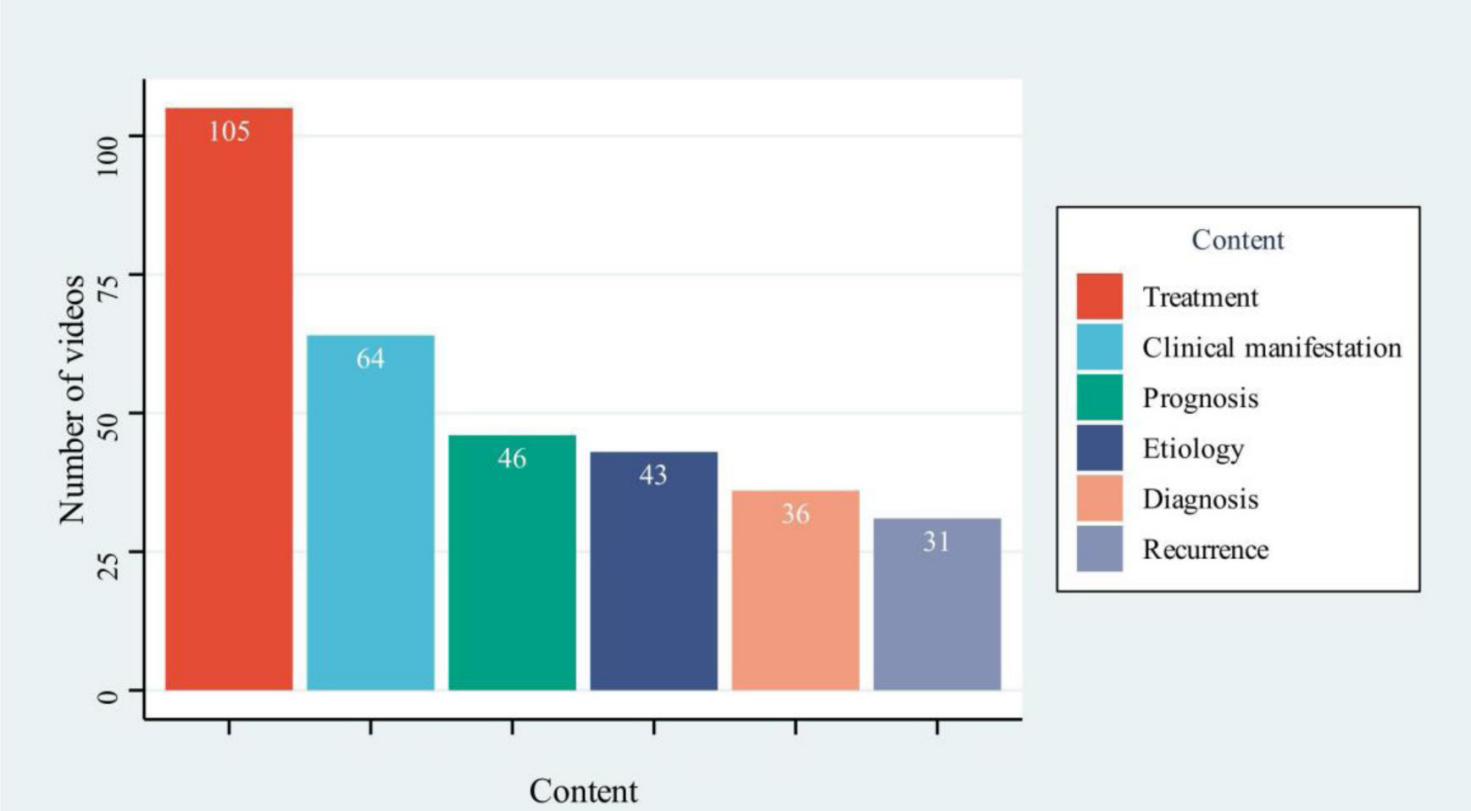
Content distribution of keloid-related videos.

Treatment (86.07%) and clinical manifestation (52.46%) dominated the content, whereas etiology, prognosis, diagnosis, and recurrence received minimal coverage (Fig. 3).

### 3.4. Differences among uploaders in video quality and reliability

**Figure 4.**
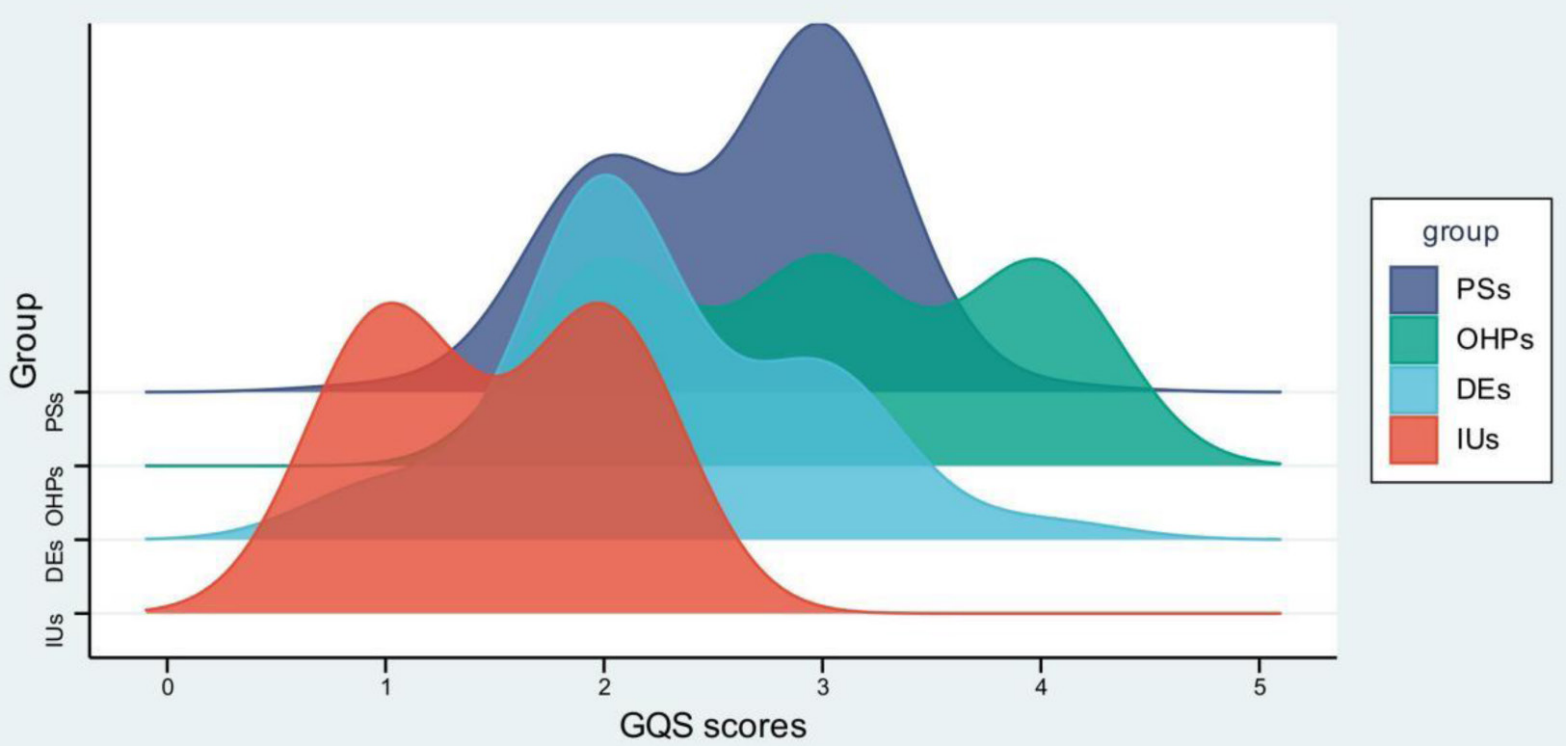
GQS score distributions across uploader groups. DEs = dermatologists, GQS = global quality score, IUs = individual users, OHPs = other healthcare professionals, PSs = plastic surgeons.

**Figure 5.**
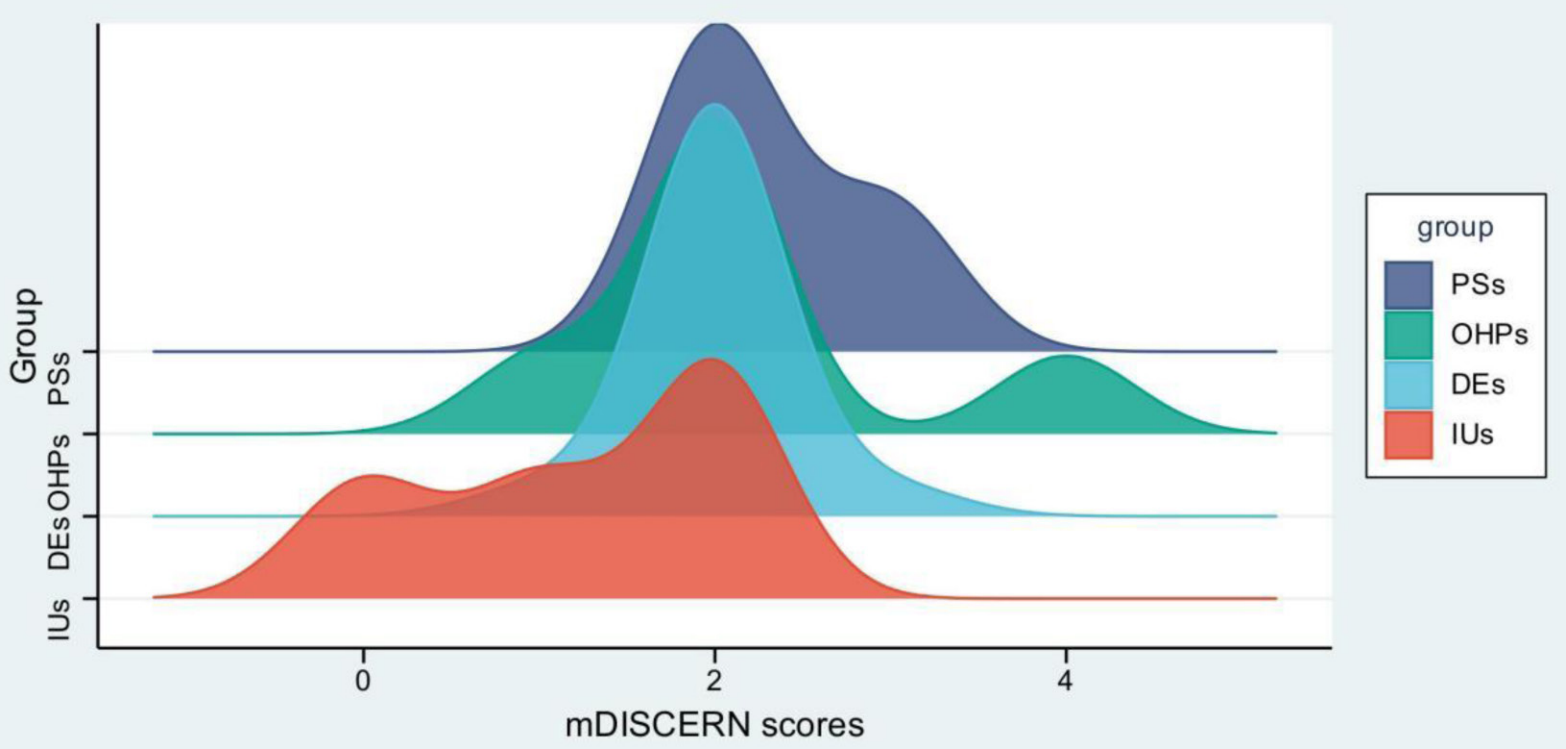
mDISCERN score distributions across uploader groups. DEs = dermatologists, IUs = individual users, mDISCERN = modified DISCERN, OHPs = other healthcare professionals, PSs = plastic surgeons.

**Figure 6.**
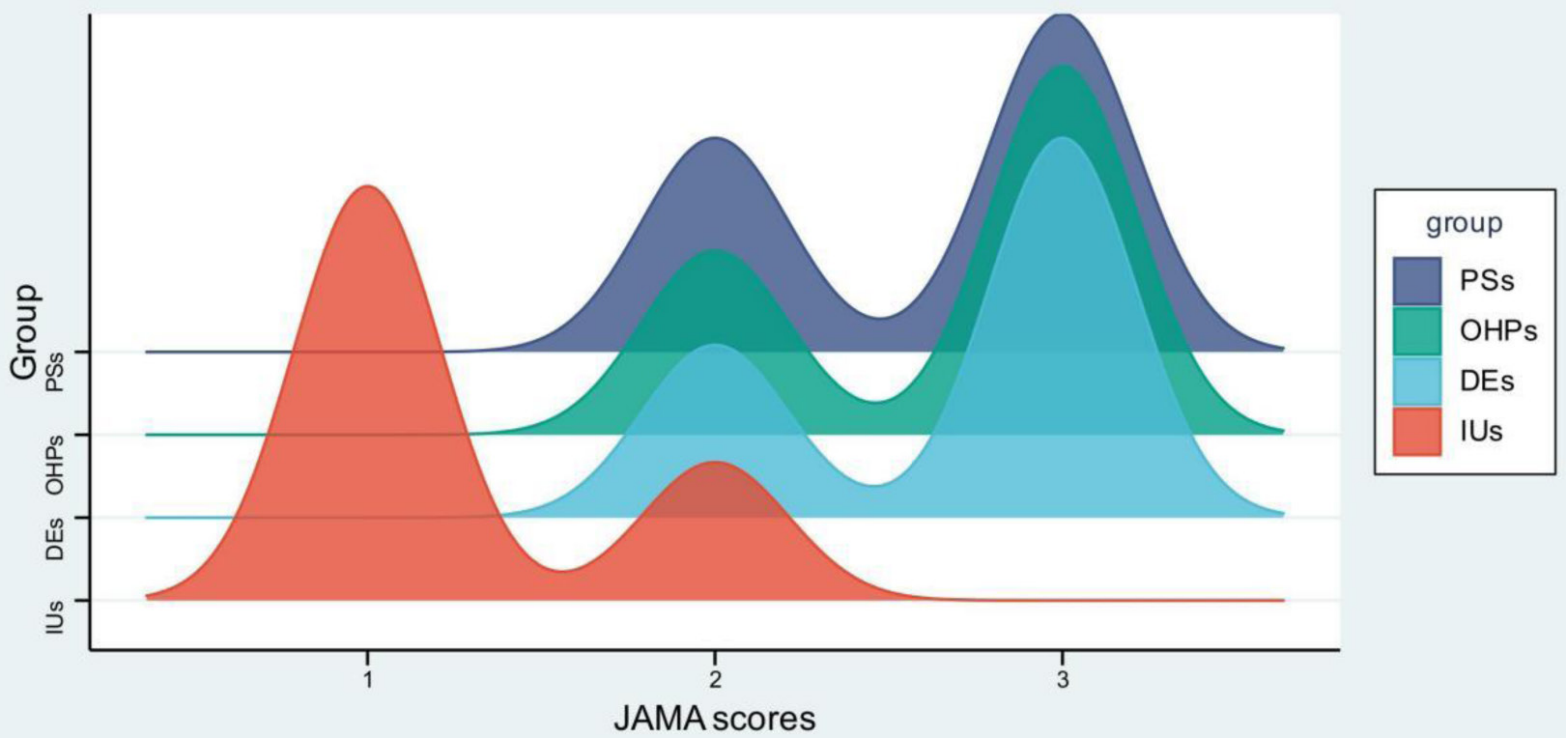
JAMA score distributions across uploader groups. DEs = dermatologists, IUs = individual users, JAMA = Journal of the American Medical Association, OHPs = other healthcare professionals, PSs = plastic surgeons.

**Figure 7.**
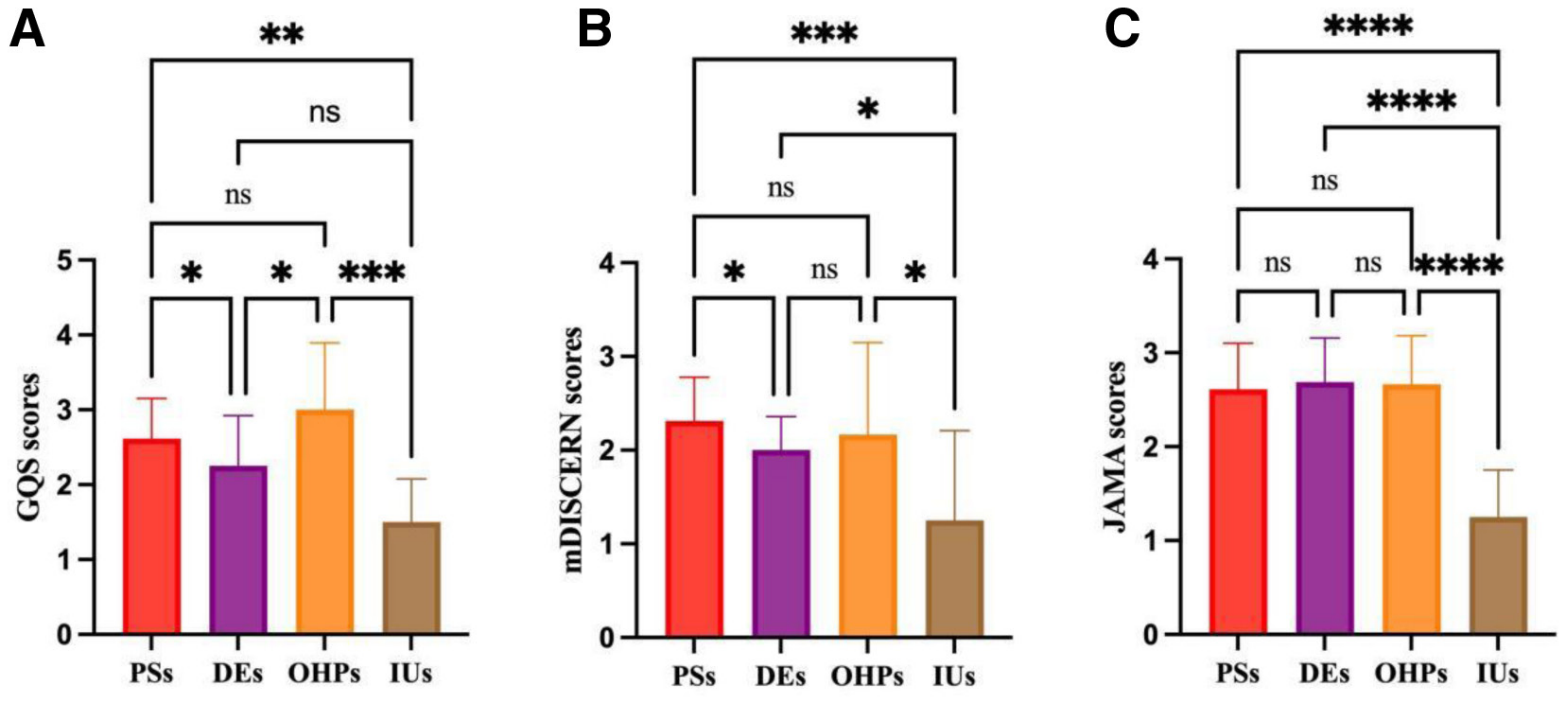
GQS, mDISCERN, and JAMA score comparisons across uploader groups (A) GQS score differences between uploader categories. (B) mDISCERN score differences between uploader categories. (C) JAMA score differences between uploader categories. * indicates *P* < .05, ** indicates *P* < .01, *** indicates *P* < .001, **** indicates *P* < .0001. DEs = dermatologists, GQS = global quality score, IUs = individual users, JAMA = Journal of the American Medical Association, mDISCERN = modified DISCERN, ns = not significant, OHPs = other healthcare professionals, PSs = plastic surgeons.

Figures 4 to 6 illustrate the variation in quality, reliability, and content scores (GQS, mDISCERN, and JAMA) across different uploader categories. Statistically significant differences were observed across groups for all 3 quality scores (*P* < .01). In pairwise analyses, material produced by plastic surgeons and OHPs attained significantly higher GQS scores compared with videos posted by individual users (Fig. 7A). Videos from plastic surgeons, dermatologists, and OHPs had significantly higher mDISCERN and JAMA scores than those from individual users (Fig. 7B and 7C). Detailed scoring parameters are shown in Table 6.

**Table 6:** Differences in video characteristics and quality among plastic surgeons, dermatologists, other healthcare professionals, and individual users.

Variables	DEs (n = 32)	IUs (n = 4)	OHPs (n = 6)	PSs (n = 80)	*P*
Video length, M (Q_1_, Q_3_)	52.00 (45.00, 71.25)	143.50 (83.75, 184.25)	80.50 (77.00, 88.50)	67.50 (42.50, 119.25)	.241
Likes, M (Q_1_, Q_3_)	184.50 (90.50, 529.50)	2126.00 (173.75, 4500.75)	144.50 (83.25, 337.00)	220.00 (91.50, 575.50)	.537
Collections, M (Q_1_, Q_3_)	54.00 (29.00, 177.50)	251.50 (40.00, 506.50)	28.50 (8.00, 110.50)	86.00 (30.50, 232.50)	.438
Comments, M (Q_1_, Q_3_)	28.00 (10.00, 104.50)	450.50 (91.50, 1003.50)	17.50 (15.50, 42.00)	20.00 (6.75, 93.00)	.124
Shares, M (Q_1_, Q_3_)	66.00 (35.25, 186.00)	483.00 (19.50, 2869.75)	34.00 (12.25, 153.25)	90.00 (24.00, 282.00)	.664
GQS, M (Q_1_, Q_3_)	2.00 (2.00, 3.00)	1.50 (1.00, 2.00)	3.00 (2.25, 3.75)	3.00 (2.00, 3.00)	<.001
mDISCERN, M (Q_1_, Q_3_)	2.00 (2.00, 2.00)	1.50 (0.75, 2.00)	2.00 (2.00, 2.00)	2.00 (2.00, 3.00)	<.001
JAMA, M (Q_1_, Q_3_)	3.00 (2.00, 3.00)	1.00 (1.00, 1.25)	3.00 (2.25, 3.00)	3.00 (2.00, 3.00)	.003

DEs = dermatologists, IUs = individual users, GQS = global quality score, JAMA = Journal of the American Medical Association, mDISCERN = modified DISCERN, OHPs = other healthcare professionals, PSs = plastic surgeons.

### 3.5. Association of engagement metrics with information quality and reliability

**Figure 8.**
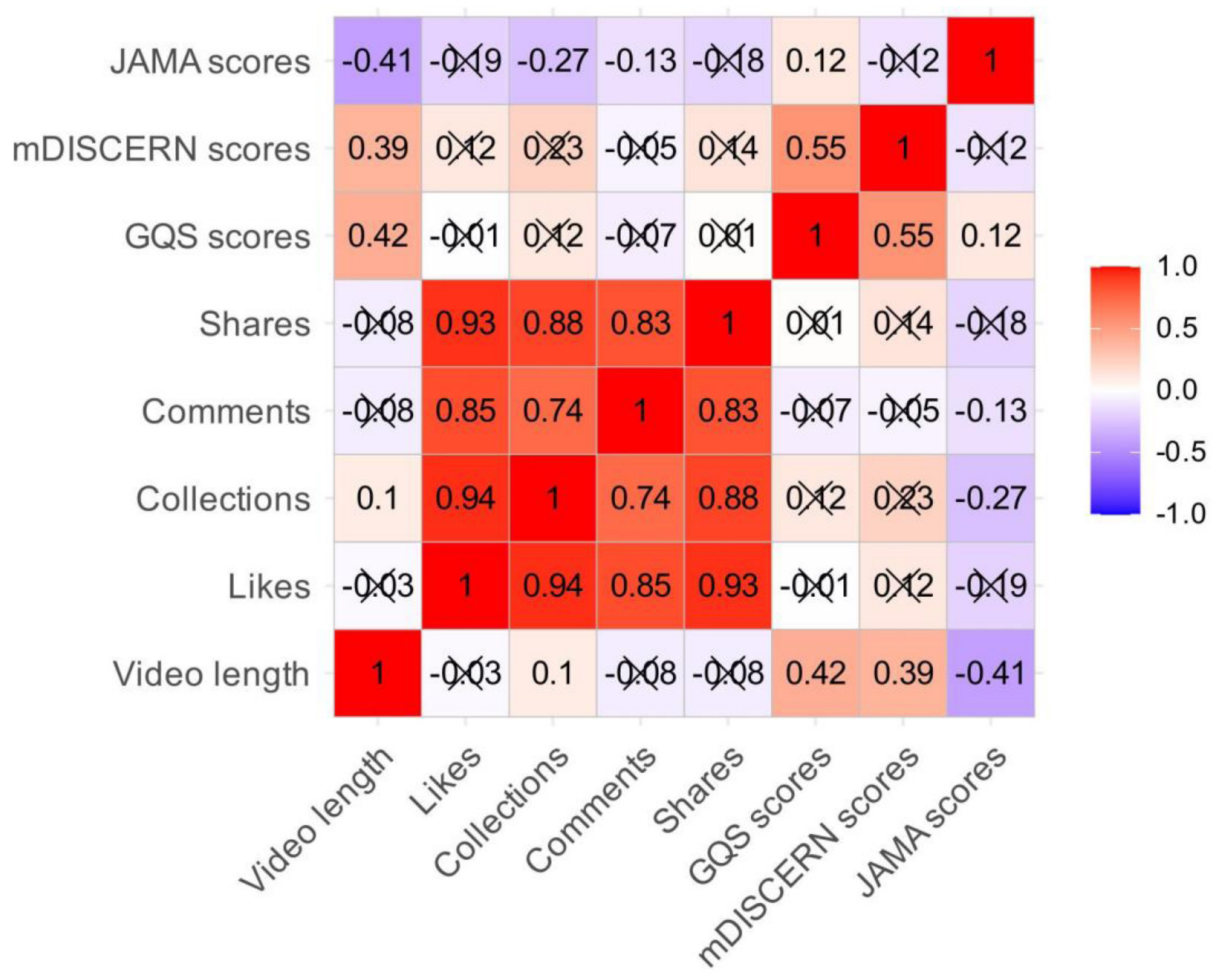
Correlation analysis heat map. GQS = global quality score, JAMA = Journal of the American Medical Association, mDISCERN = modified DISCERN.

Video length exhibited an overall moderate positive association with GQS and mDISCERN ratings, along with a weak inverse link to the JAMA score. Strong intercorrelations emerged among engagement indicators – likes, comments, shares, and collections. Nevertheless, these engagement metrics showed no significant relationship with any quality measure. A moderately robust positive correlation was observed between the GQS and the mDISCERN instruments. No additional meaningful associations were identified (Fig. 8).

## 4. Discussion

As far as we are aware, this investigation represents the inaugural systematic appraisal of the caliber of video materials and information about keloid available on TikTok, the predominant short-video platform in China. Given the substantial public health burden posed by keloid, our findings offer meaningful knowledge and support ongoing initiatives to raise population-wide consciousness about this disease.

TikTok is a powerful medium for disseminating medical expertise.^[18]^ Social media platforms provide patients with easy access to medical knowledge and avenues for seeking social support.^[19]^ Our investigation systematically evaluated the subject matter, standard, and credibility of 122 TikTok short clips concerning keloid. Findings indicated that despite garnering considerable viewer interest, the overall informational quality and trustworthiness of these materials remained notably inadequate. Therapy and clinical presentation dominated the content, whereas aspects like diagnosis and disease relapse received markedly less coverage. Crucially, videos posted by healthcare practitioners – plastic surgeons in particular – attained substantially superior GQS, mDISCERN, and JAMA ratings relative to content produced by lay individuals. At the same time, engagement indicators, including likes, remarks, and reposts, displayed no meaningful association with video quality. These results highlight a critical necessity to strengthen expert contribution and tighten content governance in the context of conveying health knowledge across social networking sites.

### 4.1. Analysis of video baseline characteristics and engagement

According to the analysis, TikTok clips addressing keloid typically demonstrate short durations, with a median of 65.50 seconds (IQR: 44.25–105.50), reflecting the broad tendency toward brevity on this platform. Such abbreviated videos satisfy the widespread user desire for quick information intake, consequently enabling rapid dissemination of knowledge.^[20]^ Despite their brevity, these videos demonstrated strong interactivity, as evidenced by high median numbers of likes, comments, saves, and shares, suggesting they are effective in engaging viewers and promoting interaction.^[21]^ However, these videos were found to have limited quality and reliability.^[22]^ The median GQS and mDISCERN scores were relatively low, at 3.00 (2.00, 3.00) and 2.00 (2.00, 2.00), respectively. Only a tiny fraction of the videos obtained flawless scores on all 3 assessment instruments, revealing that the vast majority fell short of rigorous benchmarks for both medical veracity and general excellence. Although these videos demonstrated substantial user engagement, the health-related content often proved inaccurate, fragmentary, or devoid of citations from credible sources, thereby risking the dissemination of skewed health messages to audiences. These findings are consistent with research by Zhang et al^[23]^ on osteoporosis-related TikTok videos, which also demonstrated low video quality. Another study evaluating the quality of hypertension-related videos on TikTok similarly noted that videos on this platform are of low quality and insufficient reliability, highlighting a need for improvement.^[24]^ Consequently, even though the short-video medium constitutes an efficient vehicle for health promotion, its utility is undercut by suboptimal content standards; thus, both medical fidelity and overall informational quality demand substantial refinement.

### 4.2. Differences in video quality among different uploaders

Significant differences were observed in video quality among various types of uploaders. Regarding GQS scores, videos uploaded by plastic surgeons and OHPs received higher scores than those uploaded by individual users. Such a correlation strongly indicates that the uploader’s domain-specific credentials are pivotal to safeguarding both clinical veracity and instructional quality. In terms of mDISCERN and JAMA scores, plastic surgeons, dermatologists, and OHPs all achieved relatively high scores, significantly outperforming individual users. Leveraging their expertise and clinical practice, these groups are more inclined to provide well-structured and evidence-based information. In this context, OHPs primarily refer to nuclear medicine physicians and representatives from hospitals or departments, all capable of providing professional scientific education on keloid knowledge. Comparable trends have been documented across diverse health communication studies, reinforcing that content developed by credentialed clinicians consistently exceeds user-generated material in overall quality. One investigation examining short videos on gender-affirming procedures from 2 major platforms confirmed that physician-produced videos attained significantly better ratings on validated evaluation instruments than content originating from lay contributors.^[25]^ Comparable conclusions were reached in studies concerning premature ovarian insufficiency,^[26]^ cataracts,^[27]^ and systemic lupus erythematosus.^[28]^ Nevertheless, content produced by clinically qualified physicians similarly failed to achieve exemplary benchmarks, indicating that disciplinary expertise alone cannot ensure effective knowledge translation within digital ecosystems. Formal instruction in digital dissemination – incorporating techniques for condensing evidence-based messages, securing engagement from defined audience segments, and deploying rigorous visual presentation – may empower medical practitioners to develop materials that reconcile scholarly accuracy with broad public comprehension.

### 4.3. The scope of video content

Regarding the subject matter covered, our data indicated that treatment and clinical manifestations constituted the most commonly occurring themes. Given that treatment modalities and clinical presentations are crucial for disease prevention and control, disseminating such information helps raise public awareness of the condition and facilitates better treatment outcomes.^[29]^ However, topics such as etiology, diagnosis, prognosis, and recurrence were mentioned less frequently. This indicates that while the public may have some understanding of keloid, their knowledge regarding important aspects like causes, identification methods, and recurrence prevention remains insufficient. This could lead to delays in early diagnosis and timely intervention, ultimately compromising treatment outcomes. It might also foster misconceptions about the possibility of a complete cure, potentially increasing the risk of recurrence.^[30]^ Consequently, subsequent short-video productions ought to emphasize the etiology, diagnosis, and prognosis of keloid, thereby equipping audiences with complete, evidence-based, and actionable health knowledge.

### 4.4. Correlation between engagement metrics and video quality and reliability

This study observed significant positive correlations among engagement metrics such as likes, comments, shares, and saves, suggesting that viewers’ interactive behaviors tend to occur concurrently. Nonetheless, no substantial associations emerged between the user engagement indicators and the GQS, mDISCERN, and JAMA benchmarks, demonstrating that heightened audience interaction does not inherently signify superior content quality.^[27,31]^ In addition, the analysis revealed a modest positive association between clip duration and the GQS and the mDISCERN ratings. This finding implies that lengthier videos could bolster instructional worth and caliber by delivering more exhaustive, organized, and logically coherent material, accompanied by appropriate citations. Nevertheless, merely prolonging recording time does not inherently guarantee superior quality. High-quality videos must ensure that the knowledge presented is sufficiently professional and accurate, highly relevant to the topic, and easily comprehensible for the target audience.

### 4.5. Implications of the study

Our findings demonstrate that while keloid-related short clips on the TikTok platform garnered considerable audience interaction, substantial scope remains for advancing their overall caliber and the health information they deliver. To amplify the clinical utility and communicative impact of these materials, subsequent video production should prioritize refinements across several pivotal dimensions. Initially, broader involvement of healthcare practitioners in content generation ought to be promoted, thereby strengthening the factual precision and trustworthiness of the presented knowledge. Professional involvement is particularly critical in patient education for common diseases. Second, content creators should strive to provide more comprehensive disease-related information, moving beyond introductions focused on a single aspect. Videos should systematically cover treatment, etiology, epidemiology, clinical manifestations, diagnosis, and prognosis to help the public establish a more complete understanding of the disease. Moreover, sustaining high audience interaction must coexist with a rigorous commitment to clinical accuracy and equitable presentation of information, refraining from pursuing superficial attractiveness at the cost of substantive caliber. By seamlessly merging domain expertise with captivating delivery techniques, short-video platforms could assume a markedly amplified function in advancing population health literacy.

This study has several limitations. First, while TikTok stands as China’s preeminent short-form video platform, the extent to which our results can be generalized to alternative social networking platforms remains unclear. Future research should investigate different video-sharing platforms, such as YouTube and Bilibili, to ascertain if comparable patterns emerge via cross-group comparisons, thereby enabling more comprehensive and effective health promotion across social media. Second, the comparatively limited sample size (122 videos overall) could restrict the external validity of our findings. Specifically, only 6 and 4 videos were contributed by OHPs and individual users, respectively, which restricts the reliability of quality comparisons across different uploader categories. Future studies with larger sample sizes could strengthen the dependability andtransferability of the study conclusions. Finally, our quality and trustworthiness evaluations relied on GQS, mDISCERN, and JAMA scores. Although all 3 assessment tools have gained broad application in academic practice, they inevitably involve some degree of subjectivity. Future research could incorporate larger, multilingual datasets, employ more objective quality assessment tools, and further investigate the actual impact of such videos on the public’s knowledge regarding keloid.

## 5. Conclusion

This study provides the first comprehensive assessment of content standards and information trustworthiness for keloid-themed short clips hosted on TikTok. Our findings demonstrate that while these videos attract substantial audience interaction, their overall informational quality and credibility remain suboptimal. No meaningful association was detected between engagement indicators and content performance. Materials shared by medical practitioners, especially plastic surgeons, achieved notably higher scores across all rating systems than contributions from individual users, underscoring the vital importance of specialized expertise in health communication. Furthermore, video content predominantly focused on treatment and clinical manifestations, while coverage of key topics such as etiology, diagnosis, and prognosis was insufficient. To maximize the educational utility of short-form videos in public health, it is imperative to increase healthcare practitioner participation, refine content organization, and develop stricter content oversight systems.

## Acknowledgments

We extend our sincere thanks to all individuals who took part in this research.

## Author contributions

**Conceptualization:** Honggang Li, Xuanfen Zhang.

**Data curation:** Honggang Li.

**Formal analysis:** Honggang Li.

**Investigation:** Honggang Li, Qinyuan Wang, Xuanfen Zhang.

**Methodology:** Honggang Li.

**Project administration:** Xuanfen Zhang.

**Supervision:** Xuanfen Zhang.

**Writing – original draft:** Honggang Li.

**Writing – review & editing:** Xuanfen Zhang.
